# CD56-Positive NK Cells and CD138-Positive Plasma Cells in Basal Decidua of Term Placentas in Singleton Pregnancies After Assisted Reproductive Technology Treatment of Endometriosis-Related Infertility

**DOI:** 10.3390/life15020240

**Published:** 2025-02-05

**Authors:** Stipe Dumancic, Marinela Bakotin Jakovac, Marko Drazen Mimica, Sandra Zekic Tomas, Jelena Marusic

**Affiliations:** 1 Department of Obstetrics and Gynecology, University Hospital Center Split, Spinčićeva 1, 21000 Split, Croatia; stipe.dumancic@gmail.com (S.D.); mdmimica@kbsplit.hr (M.D.M.); 2 Polyclinic Hormona, Rooseveltova 54, 21000 Split, Croatia; 3 University Department of Health Studies, University of Split, Ruđera Boškovića 35, 21000 Split, Croatia; 4 Pathology Department, University Hospital Center Split, Spinčićeva 1, 21000 Split, Croatia; sandra.zekic-tomas@mefst.hr; 5 School of Medicine, University of Split, Šoltanska 2A, 21000 Split, Croatia

**Keywords:** endometriosis, infertility, assisted reproductive technology, natural killer cells, plasma cells, basal decidua, immunohistochemistry

## Abstract

A eutopic endometrium in endometriosis shows altered immune responses, including abnormalities of NK cells and expression of plasma cells, related to reproductive issues. This study investigated the counts of CD56-positive NK cells and CD138-positive plasma cells in the basal decidua of term placentas in singleton pregnancies after endometriosis-related infertility conceived by assisted reproductive technology (ART). This single-center, case-control study involved immunohistochemical analysis of CD56-positive NK cells and CD138-positive plasma cells in basal decidua using primary monoclonal mouse antibodies, followed by secondary antibodies using a standardized protocol. CD56 and CD138 immunohistochemically positive cells were reported as the total cell count for each studied antibody expressed per 1 mm^2^ of basal decidua (Olympus BX46 and Olympus Image Analyzer). Placental samples containing basal decidua from 36 participants with endometriosis-related infertility who conceived by ART, 31 participants with male factor infertility who conceived by ART and 40 healthy controls were included. Endometriosis decidua showed the lowest median count of CD56-positive NK cells (11.5 / mm^2^, *p* = 0.039) in BD compared to male factor group (25 / mm^2^) and healthy controls (24.5 / mm^2^). No differences were found for CD138-positive plasma cells counts between study groups. Basal decidua in pregnancies after endometriosis-related infertility showed reduced total count of CD56-positive NK cells, without differences in the CD138-positive plasma cell counts compared to control groups. Future studies should investigate how changes in NK cells throughout pregnancy affect the development of perinatal complications and placental pathologies in women with endometriosis, which could uncover potential diagnostic and therapeutic targets.

## 1. Introduction

Endometriosis is a chronic, estrogen-dependent condition in which endometrial-like tissue grows outside of the uterus, often affecting the ovaries (endometriomas) or the peritoneal surfaces as superficial or deep infiltrating endometriosis [1]. In addition to cyclic pain, endometriosis is a leading cause of female infertility. Endometriosis negatively influences ovarian reserve and causes alterations in endometrial embryo receptivity [2]. Evidence indicates that endometriosis increases the risk of pregnancy complications, including miscarriage, preterm birth, placenta *praevia* and Cesarean delivery [3,4]. Studies have also reported a negative impact of endometriosis on placental pathology, including the development of endometriosis-like lesions of parietal decidua, placenta *praevia*, and inflammatory and vascular placental lesions [5,6,7]. Our recent study showed a higher occurrence of shorter placental cords, increased syncytial knotting and vascular malperfusion lesions in term placentas from pregnancies after endometriosis-related infertility conceived via assisted reproductive technology (ART) treatment, compared to placentas from pregnancies after male factor infertility and spontaneous pregnancies [8].

The immune theory links endometriosis to immune dysfunctions that impair the clearance of ectopic endometriotic lesions [9,10]. In the eutopic endometrium, abnormalities in natural killer (NK) cells and plasma cells have also been observed [10,11].

Uterine NK (uNK) cells are key elements of innate cell immunity and one of the primary cell populations in the decidualized endometrium. Contrary to CD56-negative CD16-positive peripheral NK (pNK) cells that are granular and cytotoxic, CD56-positive CD16-negative uNK cells exhibit low cytotoxicity, which is important for the immune tolerance of the embryo during implantation. Successful pregnancy depends on the processes of decidualization, embryo implantation and placentation, and NK cells play a crucial role in these phases [12]. Studies have shown increased infiltration of uNK cells during the secretory phase of the menstrual cycle and an elevated proportion of cytotoxic uNK cells marked by CD16 in patients with endometriosis and associated recurrent miscarriages [11,13].

Endometrial stromal plasma cells, marked by CD138, are involved in the defense against intrauterine infections. Chronic endometritis, characterized by CD138-positive plasma cells in the endometrium, is associated with reproductive disorders, including infertility, implantation failure and miscarriage [14]. Studies have shown an increased prevalence of chronic endometritis in patients with endometriosis, with the correlation of the CD138-positive plasma cell count in endometrial biopsies and the progression of endometriosis-related infertility [14,15]. Furthermore, plasma cells are extensively involved in immune responses in the lymph nodes of the uterine drainage pathway and endometriotic lesions and are found in greater numbers in ovarian endometriomas [16,17].

These immune alterations may drive a pro-inflammatory environment within the decidua that negatively impacts embryo development and hinders proper vascularization and placentation, resulting in pregnancy complications [18]. However, data regarding cell immunity of decidua in endometriosis are missing. The primary objective of this research was to investigate the count of CD56-positive NK cells and CD138-positive plasma cells in basal decidua, as well as their association with the placental histopathology of term placentas in singleton pregnancies after endometriosis-related infertility conceived via ART.

## 2. Materials and Methods

### 2.1. Study Participants

This single-center case–control study was carried out at the University Hospital Center Split (UHC Split) from January 2022 to March 2024. Infertile women who conceived via ART at the Department of Obstetrics and Gynecology during this period were evaluated for eligibility. Participants were excluded from the assessment if they have presented with infertility due to idiopathic infertility, polycystic ovarian syndrome (PCOS), diminished ovarian reserve (DOR), anatomic factors, oncofertility, Fragile X syndrome, or have suffered from recurrent pregnancy loss, autoimmune conditions (Hashimoto’s thyroiditis, rheumatoid arthritis, inflammatory bowel diseases), thrombophilia or other chronic non-communicable conditions (diabetes mellitus, hypertension, liver diseases). Pregnancies involving oocyte donation were also excluded.

Participants included in the endometriosis group had either laparoscopically confirmed and treated endometriosis lesions or ovarian cysts with an ultrasound appearance consistent with endometriomas without surgical treatment [19]. Participants included in the male factor group conceived via ART techniques but were otherwise healthy. We monitored the included ART pregnancies until delivery. Multiple pregnancies or those with pregnancy complications (such as miscarriage, Rh immunization, chromosomal or congenital disorders or preterm birth) were also excluded from the study. To control for the ART factor, we included healthy women with spontaneous pregnancies by the simple random sampling method (lottery method) upon their admission to the delivery department for term labor following an uneventful pregnancy (healthy control group).

The study was reported following the Strengthening the Reporting of Observational Studies in Epidemiology (STROBE) guidelines [20].

### 2.2. ART Treatment, Pregnancy and Labor

Infertile women conceived through ART treatments, including conventional in vitro fertilization (IVF) with fresh or frozen embryo transfer (FET) or intracytoplasmic sperm injection (ICSI) techniques. Controlled ovarian hyperstimulation (COH) was carried out using a starting dosage regime of 150–300 international units (IU) of recombinant human follicle-stimulating hormone (r-hFSH) within gonadotropin-releasing hormone (GnRH) antagonist protocols, while human chorionic gonadotropin (hCG) shots were used for follicular maturation and ovulation trigger according to the Department’s standard practice.

### 2.3. Immunohistochemical Analysis of Basal Decidua

The protocol for collection and sample preparation of placentas was reported in our previous study of endometriosis placental histopathology [8]. The placental preparation and immunohistochemical (IHC) analysis of the basal decidua of term placentas were performed in the Pathology department of UHC Split by one senior perinatal pathologist blinded for the assigned study group. The following protocol for immunohistochemical analysis on serial sections for each placental sample was used: paraffin sections were mounted on super frost slides (Thermo Scientific, Schwerte, Germany) and processed in an autostainer (Ventana Bench Mark Ultra Autorstainer, Ventana Roche, Tucson, AZ, USA). For the immunohistochemical detection of CD56-positive NK cells and CD138-positive plasma cells in basal decidua, we used primary monoclonal mouse antibodies (CD138/syndecan-1/B-A38; CD56/MRQ-42/RO, Ventana Rosche, Tucson, AZ, USA), followed by secondary antibodies in the Universal Dab detection kit (Ventana Roche, Tuscon, AZ, USA) as standardized protocol. For all primary antibodies, diffuse brown membranous and cytoplasmic staining was considered a positive reaction, while a sample of normal colonic mucosa served as a positive control. The IHC detection of CD56-positive NK cells and CD138-positive plasma cells in basal decidua was assessed using an Olympus BX46 microscope (Olympus Corporation, Tokyo, Japan) and Olympus Image Analyzer (Olympus Corporation, Tokyo, Japan). CD56 and CD138 immunohistochemically positive cells were reported as the total cell count for each studied antibody expressed per 1 mm^2^ of basal decidua.

### 2.4. Study Outcomes

We hypothesized that the basal decidua of term placentas from endometriosis pregnancies has lower cell counts of CD56-positive NK cells and CD138-positive plasma cells. Primary outcomes of this study are differences in cell counts of CD56-positive NK cells and CD138-positive plasma cells in the basal decidua of endometriosis placentas compared to those from pregnancies after male factor infertility and spontaneous pregnancies, quantified by total cell counts per 1 mm^2^ of basal decidua (Olympus BX46 and Olympus Image Analyzer). The secondary outcome is the relationship of CD56-positive NK cell and CD138-positive plasma cell counts in basal decidua to pathological placental lesions of term placentas.

### 2.5. Statistical Analysis

To compare the differences in counts of CD56-positive NK cells and CD138-positive plasma cells in the basal decidua of term placentas across three groups, we calculated the required sample size of a minimum of 91 placentas using G*Power 3.1, given an alpha level (α) of 0.05, power (1 − β) of 90% and large effect size (w = 0.5). The normality of data distribution was assessed using the Kolmogorov–Smirnov test, and the continuous data were reported as mean and standard deviation (SD) and median with interquartile range (IQR) between the 25th and 75th percentile. Categorical data were presented as frequency (n) and proportions (%). Group differences were analyzed using the chi-squared test (χ^2^) for categorical variables or Welch’s analysis of variance (ANOVA) with Tukey’s post hoc test for continuous variables. Receiver operating characteristics (ROC) curve analysis was conducted to examine the relationship between CD56-positive NK cell and CD138-positive plasma cell counts in the basal decidua and the histopathologic characteristics of term placentas according to the study group. Data regarding the histopathologic characteristics of term placentas were extracted from our previous study, which investigated endometriosis placental lesions [8]. The *p* value of 0.05 was set as statistically significant, while the GraphPad Prism version 10.2.3 (GraphPad Software, Boston, MA, USA) was used to perform statistical analysis.

## 3. Results

### 3.1. Participant Enrollment and Baseline Characteristics

Our recent case–control study of 107 participants investigated the histopathologic characteristics of term placentas in singleton pregnancies in women with endometriosis-related infertility. There, we described the enrollment process and baseline characteristics of the included participants [8]. Based on inclusion and exclusion criteria, 36 participants with endometriosis-related infertility who conceived by ART (ART Endometriosis), 31 participants with male factor infertility who conceived by ART (ART MF) and 40 healthy controls were included in this study. As this research was carried out on basal decidua samples from the same placentas as in the prior study, only the most important baseline characteristics were depicted here. Participants in the ART groups were significantly older compared to the healthy controls (*p* < 0.001), with a higher proportion of older primiparas (*p* < 0.001) and those with secondary infertility (*p* < 0.001). The endometriosis group had significantly longer infertility compared to the MF group (*p* < 0.001) and had a higher proportion of underweight women compared to other groups (*p* < 0.017). Sixty-one (61.1%) percent of endometriosis participants underwent excision surgery, while 38.9% received ART treatment without surgery [8].

### 3.2. CD56-Positive Natural Killer Cells in Basal Decidua

**Figure 1:**
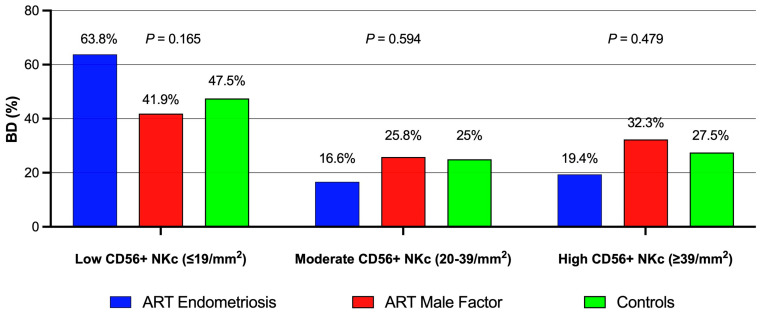
Proportions (%) of CD56-immunostained basal decidua (BD) samples across CD56-positive NK cell (CD56+ NKc) density ranges: low (≤19 / mm^2^ BD), moderate (20–39 / mm^2^ BD) and high (≥39 / mm^2^ BD). Data are categorized by study groups. ART: assisted reproductive technology. χ^2^ test, *p* < 0.05.

The results of the IHC analysis of CD56-positive NK cells (NKcs) in the basal decidua of term placentas across study groups are reported in Table 1 and Figure 1. Comparable proportions of CD56-immunostained basal decidua were observed across all groups. However, the placentas of endometriosis participants exhibited the lowest median count of CD56-positive NK cells (11.5 / mm^2^, *p* = 0.039) in basal decidua compared to the MF group (25 / mm^2^) and the controls (24.5 / mm^2^). The CD56-positive NK cell counts in the basal decidua were stratified into three density ranges: low (0–19 NKc/mm^2^), moderate (20–39 NKc/mm^2^) and high (≥40 NKc/mm^2^). Placentas from the endometriosis group exhibited the highest proportion of basal decidua with low NKc density and the lowest proportions with moderate and high NKc densities. However, these differences were not statistically significant (Figure 1).

No significant difference in CD56-positive NKc counts in the basal decidua was observed between placentas from participants with endometriosis surgery and those with untreated endometriosis before pregnancy (median count 11.5 / mm^2^ versus 10.5 / mm^2^, Table 1).

**Figure 2:**
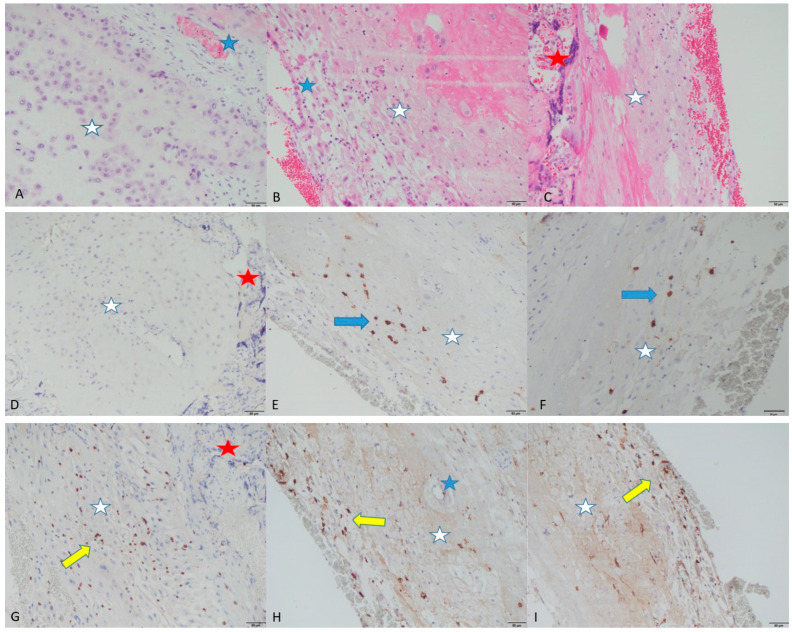
Figure plate shows images of placental slides containing basal decidua from control (**A**,**D**,**G**), ART Endometriosis (**B**,**E**,**H**) and ART Male Factor (**C**,**F**,**I**) placentas. First row images show H&E staining of placental slides, while the second and third rows demonstrate immunohistochemical CD138 and CD56 staining, respectively. White stars indicate decidua; blue stars indicate decidual blood vessels; and red stars indicate anchoring placental villi. In the images in (**E**,**F**), the blue arrow is pointing towards plasma cells, showing positive brown cytoplasmic staining for CD138, while image (**D**) shows negative CD138 immunohistochemical staining since there were no plasma cells in that section. Yellow arrows in images (**G**–**I**) indicate NK cells, showing brown cytoplasmic positive staining for CD56. Magnification for all images is 200× (Olympus Image Analyzer), and scale bar is set at 50 µm.

Figure 2G–I demonstrates CD56 immunostaining, showing brown membranous and cytoplasmic staining of NKcs in the basal decidua of control, ART Endometriosis and ART Male Factor placentas.

**Figure 3:**
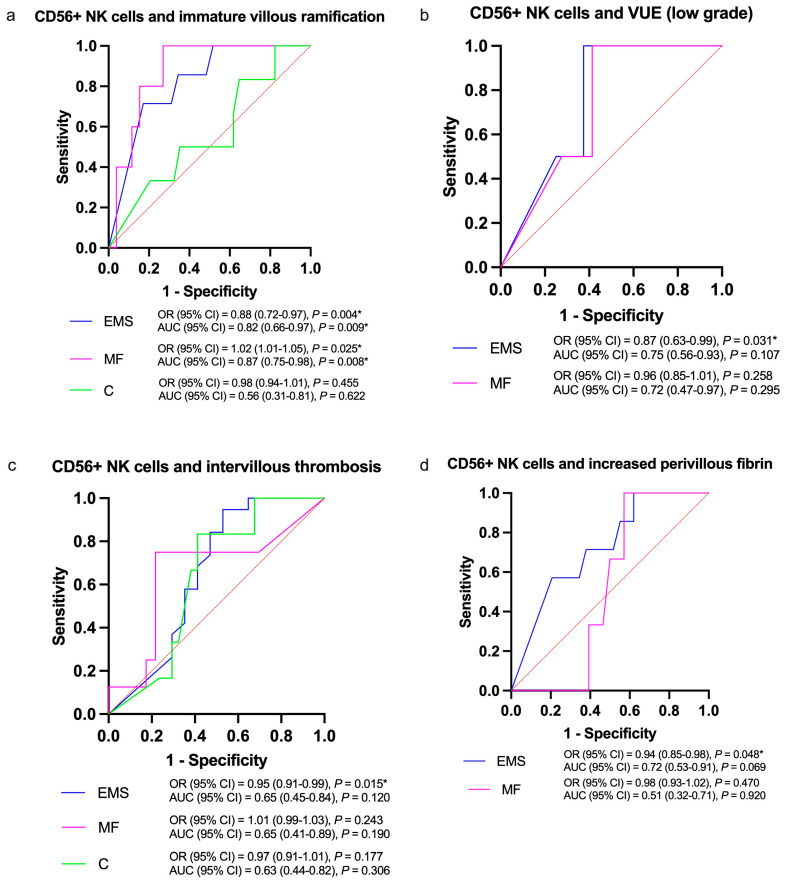
Significant odds for placental lesions with decreased CD56-positive natural killer cell (CD56+ NK) counts in the basal decidua of endometriosis placentas. Area-under-curve (AUC) and receiver operating characteristics (ROC) curves with 95% confidence intervals (CIs), while red dashed lines represent references as ROC curves of a classifiers with the random performance levels, *p* value < 0.05 (*), GraphPad Prism ver. 10.2.3. EMS: endometriosis, MF: male factor, C: healthy controls.

Regression analysis with ROC curves showed the relationship between CD56-positive NK cell counts and selected placental lesions (Figure 3a–d). We found statistically significant relations of decreased CD56+ NK cell counts in endometriosis placentas with odds for immature villous ramification (OR [95% CI] = 0.88 [0.72–0.97], *p* = 0.004), lower grade villitis of unknown etiology (VUE, OR [95% CI] = 0.87 [0.63–0.99], *p* = 0.031), intervillous thrombosis (OR [95% CI] = 0.95 [0.91–0.99], *p* = 0.015) and increased deposition of perivillous fibrin (OR [95% CI] = 0.94 [0.85–0.98], *p* = 0.048) compared to other groups, as shown in Figure 3a–d. Only the MF group showed a significant relationship of CD56+ NK cell counts with odds for immature villous ramification (OR [95% CI] = 1.02 [1.01–1.05], *p* = 0.025).

### 3.3. CD138-Positive Plasma Cells in Basal Decidua

The results of the IHC analysis of CD138-positive plasma cells in the basal decidua of term placentas across study groups are reported in Table 2. Similar proportions of basal decidua samples with positive CD138 immunostaining were observed across all groups, but these proportions were lower compared to CD56-immunostained samples. No significant differences in CD138-positive plasma cell counts were found between study groups or in the endometriosis surgery subgroup analysis (Table 2).

Figure 2D–F depicts CD138 immunostaining, showing brown membranous and cytoplasmic staining of plasma cells in the basal decidua of the control, ART Endometriosis, and ART Male Factor placentas.

In contrast to findings with CD56-positive NK cells, ROC curve analysis did not reveal significant odds for placental lesions based on CD138-positive plasma cell counts in the basal decidua of term placentas across study groups.

## 4. Discussion

Currently, there is a lack of studies assessing the number of CD56-positive NK cells and CD138-positive plasma cells in clinical pregnancy beyond the involvement of these cells in implantation failures and miscarriages in women with endometriosis-related infertility. This study demonstrated a significant decrease in CD56-positive NK cell counts in the basal decidua of term placentas in women with endometriosis-related infertility compared to controls, while CD138-positive plasma cell counts showed no differences. Several factors disseminate the discussion around NK cell abnormalities in the eutopic endometrium and decidua affected by endometriosis, including NK cell dynamics in endometrium and decidua, reproductive issues related to endometriosis (recurrent implantation failures, RIF), the impact of ART and NK cell dysfunction in complicated pregnancies.

Uterine NK cells increase from the proliferative to late secretory menstrual phase in eutopic endometrium, whereas ectopic endometriotic lesions exhibit low uNK cell counts [21]. In endometriosis, menstrual effluent shows reduced uNK cells, particularly CD56-positive CD16-negative NK cells, with diminished cytotoxicity compared to pNK cells [22,23]. Endometriosis-related infertility negatively affects embryo quality and endometrial receptivity, leading to implantation failures [24]. Bioinformatic analysis has identified a decreased fraction of resting NK cells and an increased fraction of activated NK cells in the eutopic endometrium during severe endometriosis (r-AFS III/IV) compared to mild stages (r-AFS I/II) [25]. Additionally, the mid-secretory endometrium in implantation failures associated with endometriosis exhibited elevated cytotoxic CD16-positive uNK cells [13]. These abnormalities may contribute to the immune tolerance escape of ectopic lesions and a pro-inflammatory state that impairs successful implantation [13,25].

The cyclic nature of uNK cells suggests hormonal regulation. In normal menstrual cycles and non-endometriosis decidua, uNK cells express estrogen receptor ß (ERß) and glucocorticoid receptor (GR) but lack mRNA for ERα and progesterone receptor (PR), suggesting direct regulation by estrogen and glucocorticoids [26]. Rising progesterone levels during early pregnancy indirectly stimulate uNK cell proliferation by inducing interleukin-15 (IL-15) production in the endometrial stromal cells of the decidualized endometrium [27,28]. Endometriosis is a state of progesterone resistance, leading to impaired decidualization and epithelial proliferation under estrogen excess [29]. Eutopic endometrial stromal cells exhibit deficient progesterone-sensitive gene expression, including down-regulation of the IL-15 gene [22,30]. uNK cells, with low cytotoxicity, play a key role in embryo tolerance through interactions between NK inhibitory receptors (KIRs) and non-classical HLA molecules on extravillous trophoblast cells [31]. However, the eutopic endometrium in endometriosis shows reduced HLA-G expression, potentially impairing endometrial receptivity and decidualization and contributing to implantation failure and miscarriages [32,33]. While this study did not assess progesterone effects or PR expression, altered progesterone-mediated regulation of NK cells may contribute to the decreased CD56-positive NK cell counts.

Endometriosis, as one of the leading causes of infertility, often requires ART to improve pregnancy rates. Hormonal stimulation during COH impacts uNK cell distribution during the implantation window, reducing their pro-invasive effects during early pregnancy. Kanter et al. reported reduced CD56-positive uNK cell counts in the stimulated endometrium, impairing EVT invasion [34]. Similarly, Glover et al. found that higher CD34-positive uNK progenitor cell populations, with increased CD56 marker co-expression, may predict successful implantation in endometriosis [11]. Basal decidua in ART pregnancies with allogeneic embryos showed higher CD56-positive NK cell accumulation than those with autologous embryos, suggesting impaired maternal–fetal immune tolerance linked to NK cell dysfunction [35]. Regarding frozen embryo transfer (FET) as a common ART option, natural cycles without hormonal stimulation maintain endometrial thickness [36]. Luteal phase biopsies in infertile women before FET revealed lower CD16-positive uNK cell levels in pregnant compared to non-pregnant patients, suggesting a positive prognostic factor [37]. Although this study did not analyze specific ART techniques, we acknowledge that stimulated cycles may influence CD56-positive NK cell counts differently than FET cycles [11,34]. The use of different ART techniques (fresh and frozen ET cycles) and the associated early estrogen levels must be addressed as a potential confounding factor. Differences in estrogen levels exist between stimulated cycles with fresh embryo transfer (ET), stimulated FET cycles and FET in natural cycles, with varying levels of hyperestrogenism being associated with differences in placental development during the early stages of pregnancy [38,39]. In this study, there were only three FET cases in the natural cycle within the ART Endometriosis group and four cases in the ART Male Factor group. Ganer Herman et al. observed only a higher rate of velamentous cord insertion associated with elevated estradiol levels [39]. However, no studies have investigated the impact of early estrogen levels—potentially influencing decidualization—on CD56-positive NK cell counts in the basal decidua of term placentas. While we do not consider the included FET cases and their likely lower estrogen levels as negligible, future studies should explore the possible association between estradiol levels and CD56-positive NK cell counts throughout gestation, stratified by ART techniques.

The number of pNK cells and uNK cells decreases during uncomplicated pregnancies in women without endometriosis [40,41]. However, even in such pregnancies, CD69 expression is elevated in CD56-positive CD16-positive pNK cells in venous blood, suggesting potential cytotoxicity and target trophoblast lysis [40]. In pregnancies complicated by preeclampsia or intrauterine growth restriction syndrome (IUGR), impaired NK cell function has been observed [42,43]. Preeclampsia and endometriosis share endometrial molecular pathways, including the altered expression of differentially expressed genes (DEGs) involved in NK cell function and decidualization [42]. Similarly, IUGR, often associated with placental dysfunction, is characterized by reduced CD56-positive NK cell density in the placenta [43].

In the endometriosis group, decreased CD56-positive NK cell counts were associated with significant odds of increased syncytial knotting, low-grade VUE, intervillous thrombosis and increased perivillous fibrin deposition, findings not observed with CD138-positive plasma cell counts. The proportion of CD138-immunostained basal decidua was low across groups. Rudenko et al. reported immune alterations in allogeneic ART pregnancies compared to autologous ART pregnancies, with a significant accumulation of CD56-positive NK cells and CD-138 plasma cells in the basal decidua. These changes were accompanied by chronic placental inflammation, including chronic histiocytic intervillositis, lymphoplasmacytic deciduitis, chronic chorioamnionitis, chronic villitis and perivillous fibrinoid with lymphocytes [35].

Previous studies have not established consolidated diagnostic or prognostic values for pNK or uNK cell measurements, with inconclusive evidence on the role of uNK cell numbers in successful implantation [11]. High uNK levels have been examined in reproductive diseases, but low levels remain underexplored [12,44]. No differences were observed in the proportions of CD56-immunostained basal decidua across NK cell ranges among study groups (Figure 1). A combined immunostaining approach for CD56, CD138 and BCL-6 has shown diagnostic potential in unexplained infertility and guiding targeted therapies for reproductive failure [45]. Although reference values for CD56-positive NK cells during pregnancy remain undefined, complications related to endometrial or placental dysfunction may involve impaired NK cell expression and function. A standardized protocol for immune cell assessment in the endometrium as a clinical test is needed [11,46,47].

Potential immunomodulatory strategies targeting NK cells in endometriosis include glucocorticoids, intravenous immunoglobulins (IVIGs), intralipids [12], anti-TNFα medications (e.g., pentoxifylline and recombinant antagonists) [48], *Bacillus* of Calmette–Guerin (BCG) [49], lipiodol uterine infusion via hysterosalpingogram (HSG) [50], protopanaxadiol [51], NK cell infusion or modulation of NK cell receptors [49,52,53]. These therapies affect NK cell number and function either directly or indirectly. There is ongoing debate over whether immunotherapy should target altered uNK function rather than cell numbers, as seen in cancer immunotherapy. This is due to the complex interactions of uNK cells with other immune cells and mediators and the potential negative effects of suppressing elevated uNK levels, which could inadvertently affect other immune components [44]. Separate NK cell-based immunotherapies may be needed for ectopic endometriotic lesions and for addressing altered endometrial receptivity or pregnancy complications in endometriosis. pNK cells, characterized by their cytotoxicity, could be targeted to clear endometriotic lesions in the peritoneal cavity. In contrast, uNK cells lack cytotoxicity to promote embryo tolerance at the maternal–fetal interface, and enhancing pNK cell effects in the decidualized endometrium could have deleterious outcomes [12,44].

While endometriosis is linked to chronic endometritis, we did not investigate this condition in our participants [14,15]. There were no differences in CD138-positive plasma cell counts in the basal decidua of endometriosis placentas compared to control groups, nor any association with placental histopathologic lesions. Plasma cells are key mediators of the adaptive immune response in chronic endometritis [54], but it is unlikely that endometriosis is a direct cause of this condition [55]. Our findings suggest that CD56-positive NK cells likely play a more critical role in decidualization and placentation as part of the innate immune response necessary for successful embryo implantation [24]. Additionally, type IV graft-versus-host hypersensitivity reactions, characterized by T-lymphocytes and NK cells, do not involve plasma cells [56].

This is the first study that has revealed a reduced CD56-positive NK cell count in term decidua basalis following ART treatment for endometriosis-related infertility. These findings highlight an altered immune response in basal decidua and provide a foundation for future research on the impact of endometriosis, not only on reproduction and early pregnancy successes but also on late pregnancy outcomes and placental pathology. Study limitations include potential sampling bias, the inability to sample specimens across pregnancy stages and a small sample size. Most infertile participants in the endometriosis group were diagnosed surgically as the gold standard, while others underwent ART without surgery, following the current European Society of Human Reproduction and Embryology (ESHRE) Guidelines for managing ovarian endometriomas less than 5 cm without surgical treatment, as practiced in our Department [57]. Cell measurements in decidua samples from early pregnancy were not included, nor did we measure cell counts in peripheral blood since there are findings of altered NK cell counts in early decidua observed in pregnancy loss or implantation failures in endometriosis [13]. Early pregnancy endometrial samples that would have resulted in live births were also unavailable to us. Studies speculate that uNK cells originate from a decidualized endometrium containing progenitor NK cells but could also be developed from pNK cells that infiltrate the endometrium at late secretory endometrium and early pregnancy [21,23]. This study was not designed to explore the impact of CD56-positive NK cell counts in term decidua on implantation or early pregnancy outcomes. However, we can discuss potential mechanisms associated with the altered NK cell counts observed in endometriosis placentas. The smaller sample size, while sufficient for group comparisons, is acknowledged. We assessed all 40 women with endometriosis-related infertility who conceived via ART and had a live birth during the two-year study period at our regional reproductive medicine center in Croatia [8]. The small number of infertile women with endometriosis who achieved term live birth—amid the unknown incidence of endometriosis in Croatia—and the blinding of the senior perinatal pathologist to the study group during immunohistochemical analysis are considered strengths of this study.

## 5. Conclusions

Women with endometriosis-related infertility in this study were free from chronic diseases apart from endometriosis and experienced pregnancies without the burden of perinatal complications. However, this study identifies a decreased count of CD56-positive NK cells in the basal decidua of term placentas in pregnancies following ART treatment for endometriosis-related infertility. This finding suggests that endometriosis may influence the immune environment of the basal decidua—the maternal component of the placenta—potentially contributing to placental lesions. Future research should explore how changes in uterine and peripheral NK cells throughout pregnancy affect the development of perinatal complications, placental-related diseases and placental pathologies in women with endometriosis, potentially uncovering new diagnostic and therapeutic targets.

## Figures and Tables

**Table 1:** Counts of CD56-positive natural killer cells in basal decidua of term placentas between study groups by immunohistochemistry.

	ART Endometriosis (N = 36)	ART Male Factor (N = 31)	Controls (N = 40)	*p*
No. of basal decidua ^†^	26 (72.2)	21 (67.7)	31 (77.5)	0.652
**CD56+ NK cell count** *				
Mean (SD)	18.3 (20.6)	39.5 (47.9)	29.8 (36.1)	0.039
Median (IQR, 25–75)	11.5 (29.5, 0–29.5)	25 (55, 0–55)	24.5 (40.75, 3.5–44.25)	
**Surgery vs. No surgery** ^‡^				
Mean (SD)	16.8 (18.4) vs. 20.6 (24.2)	-	-	
Median (IQR, 25–75)	11.5 (28, 0–28) vs. 10.5 (38.5, 3–41.5)	-	-	0.682

ART: assisted reproductive technology. ^†^ Frequency and proportion (n, %) of basal decidua samples stained for CD56. * Counts per mm^2^ of basal decidua reported with mean and standard deviation (SD) or median with interquartile range (IQR, 25th and 75th percentile), analyzed by chi-squared test (χ^2^) or Welch analysis of variance (ANOVA) with Levene’s test and Tukey’s post hoc test, accordingly. *p* value < 0.05. ^‡^ Counts in basal decidua in endometriosis participants with excision surgery prior to pregnancy (n = 22) versus without surgery (n = 14).

**Table 2:** Counts of CD138-positive plasma cells in basal decidua of term placentas between study groups by immunohistochemistry.

	ART Endometriosis (N = 36)	ART Male Factor (N = 31)	Controls (N = 40)	*p*
No. of basal decidua ^†^	3 (8.3)	3 (9.7)	4 (10)	0.967
**CD138+ Plasma cell count** *				
Mean (SD)	0.3 (1.4)	0.3 (1)	2 (7)	0.326
Median (IQR, 25–75)	0 (0, 0–0)	0 (0, 0–0)	0 (0, 0–0)	
**Surgery vs. No surgery** ^‡^				
Mean (SD)	0.5 (1.7) vs. 0.1 (0.3)	-	-	0.813
Median (IQR, 25–75)	0 (0, 0–0)/0 (0, 0–0)	-	-	

ART: assisted reproductive technology. ^†^ Frequency and proportion (n, %) of basal decidua samples stained for CD138. * Counts per mm^2^ of basal decidua reported with mean and standard deviation (SD) or median with interquartile range (IQR, 25th and 75th percentile), analyzed by chi-squared test (χ^2^) or Welch analysis of variance (ANOVA) with Levene’s test and Tukey’s post hoc test, accordingly. *p* value < 0.05. ^‡^ Counts in basal decidua in endometriosis participants with excision surgery prior to pregnancy (n = 22) versus without surgery (n = 14).

## Data Availability

The datasets that support the findings of this study are available from the corresponding author upon reasonable request.
